# NMR spectroscopy of HIV-1 gp120 outer domain

**DOI:** 10.1186/1742-4690-9-S2-P20

**Published:** 2012-09-13

**Authors:** M Sastry, L Xu, S Bhattacharya, GJ Nabel, CA Bewley, PD Kwong

**Affiliations:** 1 Vaccine Research Center, NIAID/NIH, Bethesda, MD, USA; 2 New York Structural Biology Center, New York, NY, USA; 3 NIDDK, National Institutes of Health, Bethesda, MD, USA

## Background

The outer domain (OD) of HIV-1 gp120 has been proposed as a minimal immunogen to elicit broadly neutralizing antibodies. However, OD is heavily glycosylated, contains many flexible regions, and immunization with a number of different OD variants has thus far failed to elicit neutralizing antibodies. An understanding of the conformational space sampled by the OD in its unliganded state, however, may assist in the use of OD as an immunogen.

## Methods

We developed a method to isotopically enrich glycoproteins using a mammalian expression system that exploits the high level of protein expression obtained from an adenoviral vector, and employed heteronuclear NMR spectroscopy to obtain structural and dynamic information of unliganded OD. Multidimensional NMR experiments were recorded on uniformly labeled ^15N/13^C OD as well as on samples selectively enriched in ^15^N-labeled Gly, Ile, Leu and Val. Experiments for backbone assignments were also recorded on an OD sample enriched in ^15N/13^C for Ile, Leu and Val.

## Results

We successfully produced isotopically labeled OD samples, suitable for NMR analysis. We also identified Gly, Ser, Val, Leu and Ile residues using samples selectively enriched in ^15^N for Gly, Val, Leu and Ile. Standard triple resonance NMR experiments on the isotopically labeled OD were combined with backbone experiments recorded on a second sample – that was selectively enriched in ^15N/13^C for Ile, Leu and Val – to assign HN, C’, C_alpha_ and N backbone resonances in about 80 of the 220 residues of OD.

## Conclusion

We succeeded in assigning ~1/3 of the backbone for unliganded OD with triple resonance experiments. Extension of these assignments with NOESY experiments is now proceeding. Our results indicate that a solution structure of the highly glycosylated HIV-1 gp120 OD is feasible.

